# Assessment for Different Neural Networks with FeatureSelection in Classification Issue

**DOI:** 10.3390/s22083099

**Published:** 2022-04-18

**Authors:** Joy Iong-Zong Chen, Chung-Sheng Pi

**Affiliations:** Department of Electrical Engineering, Da-Yeh University, Chunghua 515006, Taiwan; d1003004@cloud.dyu.edu.tw

**Keywords:** CC (correlation coefficient), NN (neural network), FS (feature selection), supervised learning, self-revision learning

## Abstract

In general, the investigation of NN (neural network) computing systems requires the management of a significant number of simultaneous distinct algorithms, such as parallel computing, fault tolerance, classification, and data optimization. Supervised learning for NN originally comes from certain parameters, such as self-revised learning, input learning datasets, and multiple second learning processes. Specifically, the operation continues to adjust the NN connection synapses’ weight to achieve a self-learning computer system. The current article is aimed at developing the CC (correlation coefficient) assignment scheme adaptively joint with the FS (feature selection) categories to pursue the solutions utilized in solving the restrictions of NN computing. The NN computing system is expected to solve high-dimensional data, data overfitting, and strict FS problems. Hence, the Fruits-360 dataset is applied in the current article, that is, the variety of fruits, the sameness of color, and the differences in appearance features are utilized to examine the NN system accuracy, performance, and loss rate. Accordingly, there are 120 different kinds with a total of 20,860 fruit image datasets collected from AlexNet, GoogLeNet, and ResNet101, which were implemented in the CC assignment scheme proposed in this article. The results are employed to verify that the accuracy rate can be improved by reducing strict FS. Finally, the results of accuracy rate from the training held for the three NN frameworks are discussed. It was discovered that the GoogLeNet model presented the most significant FS performance. The demonstrated outcomes validate that the proposed CC assignment schemes are absolutely worthwhile in designing and choosing an NN training model for feature discrimination. From the simulation results, it has been observed that the FS-based CC assignment improves the accurate rate of recognition compared to the existing state-of-the-art approaches.

## 1. Introduction

Lectures that address data FS (feature selection) issues and NN (neural network) performance evaluation, as well as the contribution to the proposed CC (correlation coefficient) assignment algorithms, are introduced in this section.

### 1.1. General Description to the Relative Works

An NN frame, such as deep learning training and test verification, has generated many mature artificial intelligence applications, such as object detection and object classification. NN application to image recognition has gradually become a technology that provides high added value products in the market. Moreover, it can also apply to parallel computing, fault tolerance, classification, and data optimization. It is also believed that a trend must be followed in deep learning and machine learning applications in the future. What can be seen right now is that the high-precision classification and recognition produced by these deep learning systems have already produced very fruitful results. It is known that, traditionally, the application of FS is mainly to obtain great prediction performance of the NN, support more-effective algorithm of the NN framework, and provide better recognition of the underlying process that introduced the data. In other words, FS has become critical issue addressed by the researchers investigating the area in which a large number of variables are available [1]. In [2], the authors employ automatic methods for FS and study their performance in real SCADA (supervisory control and data acquisition) data, choosing the optimal and adequate number of variables related to a failure, which is a key step when making the model. In general, the selected way for processing deep learning could improve the dataset training depth. For example, when increasing data training depth, try to expose the training set object features and proceed delicately with training set preprocessing [3,4]. Therefore, most of the deep learning processes for image recognition, image classification, or object detection are expected to not be limited by the number of application parameters. In fact, there are many strategies, for instance, in a deep training environment that uses a large number of datasets, to try to create parameters that can participate in the training application as much as possible and are not limited by the training framework complexity [4]. However, most of the publications did not explore the CC assignment scheme that is a novelty focused on in the current article, which is also able to be adaptively generated by the algorithm proposed in description as in the following sections. To the best of our knowledge, the presented method is a completely fresh application to the research field of image recognition and machine learning.

Generally speaking, in the standard deep learning and machine learning process, a very large amount of datasets will be inevitably used. At this time there will be several factors that affect the training framework effectiveness. The data overfitting problem is encountered first, which is normally caused by the data training, verification, and testing processes that are not in accordance with the normal quantitative ratio. Due to the inappropriateness and lengthiness of high-dimensional data, the training framework performance deteriorates [4]. Other issues, such as improper activation function selection, ineffective planning for data validity, etc., will be reasons for learning algorithm misjudgment and training framework deterioration. As mentioned previously, the deep learning framework is nothing more than high-precision classification and identification. Many studies have published many results focusing on deeper data learning and training to improve dataset training depth. The multi-faceted and in-depth combination of training set object characteristics were published in a large number of references. A novel convolutional architecture, termed SpiderCNN, comprised of SpiderConv units is proposed to efficiently extract geometric features from point clouds.

Several traditional data extraction methods are used for classification that could provide sufficient information for further data analysis classification steps. FS is usually a skill applied to solve the data dimension problem in classification, recognition, and validation. There is an innovative learning methodology for cross-domain semantic segmentation, termed progressive feature refinement, presented in [5]. The related literature published on preprocessing the training set is relatively rare. The main reason is that it takes a long time to collect the training set, and data preprocessing has a relatively high variability, which requires more frequent and in-depth data preprocessing exploration. The related research results are even rarer [6]. The dimension reduction method plays an inevitable role in analyzing and visualizing high-dimensional multi-source data. In [7], the authors adopted quality metrics to compare the performance for the integrative analysis of multi-omic datasets from the multi-view category to evaluate benchmark 87 method performance. In [8], MICIMR (maximal independent classification information and minimal redundancy), a hybrid altered FS algorithm based on information theory, was proposed. It outperforms the other FS algorithms. In addition, classical ML (machine learning) methods [9] were applied to expert support in terms of time and effort by making sense of features. However, images may be affected by different lighting conditions during image acquisition, which causes worse performance results [10,11]. Two specific conditions are required: the network should be designed according to these features before classification, and the features to be used should be well determined [12].

Furthermore, developing applications for data preprocessing FS is a difficult task. For instance, the basic introduction and the “Order Statistics” applications are illustrated in [13,14] publications, respectively. The feature correlation has gradually become a useful method for solving image preprocessing problems. The tiny data feature (sub feature data) that distinguishes a class from a traditional class using CC and fuzzy model to select features and sub features is presented in [15]. In [16], the ways to find the hidden information in 3D stage analysis with a novel FS algorithm taking into account both feature robustness and relevance to mitigate the CSM problem is discussed. There are still huge amounts of existing reports that show deep learning NN performance can be improved by careful FS assessment [17,18]. Based on the review to the aforementioned works, the CC (correlation coefficient) assignment scheme developed in the current article for facilitating the traditional FS categories to pursue the solutions is utilized in solving the restrictions of NN computing. Furthermore, another theoretical approach relevant to the discussion of the FS issue is PCA (Principal Component Analysis). PCA is defined as a mathematical procedure which transforms a number of possibly correlated variables into a smaller number of uncorrelated. However, due to the assumptions that PCA has orthogonality and variance dependency, it can generate ill effects on the resulting principal components depending on the dataset.

In summary, the CC assignment technique proposed in this article is deployed to enhance the performance of the traditional FS categories method. Finally, the full illustrating efficiency of the proposed methodology for validating the outcome is evaluated by three CNN-based NN frameworks.

### 1.2. Contributions and Organization of this Article

Traditionally, NNs with high computing and high energy consumption have messiness and imbalance due to huge amounts of data. To avoid feeding huge amounts of training data into machine learning or deep learning algorithms, due to its high-dimensional inappropriateness and verbosity, the training framework effectiveness is likely to deteriorate. In data preprocessing, the jpdf (joint probability density function) of the CC (correlation coefficient) value of the data feature is obtained by derivation. The mpdf (marginal probability density function) of each data feature is then determined. Therefore, according to the obtained pdf, different correlation coefficient values can be assigned to the data characteristics first. Once the data characteristics with sequential correlation coefficients are classified more finely, the confusion and imbalance levels of the data can be reduced. The solution, relatively speaking, can expand the generated data feature selectivity. In other words, the data features will be distinguished and presented more significantly. After the data is preprocessed using the assigned CC value, the article uses three learning and training algorithms based on the CNN operation (convolutional neural network) to obtain the network framework. The training framework accuracy rate and loss function are analyzed first, and then validated and tested. The data is verified, and finally compared with the model analysis results generated from the traditional algorithm.

Before serious expression of the contributions for the proposed CC assignment scheme, it is worth taking a moment to deeply explore the issue addressed in this work. Based on the discussion of the relevant works in the previous Section 1.1, where the motivation of studies almost aimed at proposing algorithms with “feature extraction” or “feature selection”. Nevertheless, the impact of the huge number of datasets, the high dimension of data volume, and large tensor of data vectors has resulted in the consideration of very few solutions. Since those are critical problems to search the necessary solutions such as the novel methods proposed in the present article. Thereafter, the benefits of the proposed CC assignment include reducing the dimension of the data volume and even lowering the data vectors. Certainly, the use of the proposed CC assignment method has many starting conditions that are assumed. Firstly, different kinds of recognized images are applied except for colorful ones, and the unit dimension photo is adopted. Next, to validate the operation, the proposed novel method currently avoids adopting “manual” FS which is determined with an CC assignment, and this allows the CNN to extract the relevant features during its training. Many relevant assumptions for the preprocessing of gathered images are going to be described in the Section 4.

So far, data preprocessing to address the FS issue with the CC indication has been rare. According to the authors’ knowledge, the proposed CC value assignment method for selective feature is a brand new approach. Based on the aforementioned description, an algorithm for deriving the formulas for CC assignment to promote FS is implemented in this work. In summary, this investigation has the following contributions.

I. Solving the inappropriate problem of huge datasets with high-dimensional Neural Networks adopts traditional FS.
II. The CC assignment scheme developed with the basis of theoretical jpdf and mpdf formulas for facilitating the traditional FS method.
III. Proposing a rule of CC assignment which is exemplified in Section 3 with different kinds of NN frameworks for increasing the validation to the FS characteristics.
IV. Certificating the effectiveness of the proposed ways and increasing the benefits of FS categories by the deployment of three conventional frameworks, AlexNet, GoogLeNet, and ResNet101 which are based on the CNN model.
V. Most of the results in performance are evaluated by employing many remarkable algorithms and tools and are finally compared with existing publications.

The rest of this paper is organized with five sections as follows. After the Section 1, the problem formation describes the FS issues for machine learning or deep learning expressed in Section 2. In Section 3, a total solution is proposed with the traditional concept based on FS with CNN architecture. The models of the CC value assignment technique and the applications to the algorithm for increasing the FS of the dataset are presented in Section 4 and Section 5, respectively. The discussion of the results of the proposed algorithm and an example are given in Section 4. Finally, there is a short conclusion given in Section 5.

## 2. FS Issues

The FS terminology is an important issue addressed in NN deep training and testing. The literature survey and its relationship to machine learning and deep learning are discussed in the section.

The FS techniques are not a fresh skill adopted in the huge NN architecture computing requirement. However, in recent years, there have still been numerous published research reports that discuss such issues as selective features. In [18], the focus is on providing the big picture on filtering techniques for DEGs (Differentially Expressed Genes) in discovering a unified technical framework to outline common points and their particulars. An intrusion detection model using Chi-square FS and multi class SVM (Support Vector Machine) is proposed in [19]. In [20], the authors proposed a new FS method referred to as EFS (Extensive Feature Selector), which benefits from corpus-based and class-based probabilities in its calculations. The EFS performance is compared with nine other FS methods. The effect of globalization techniques is investigated using three LFS methods named as DFSS (Discriminative Feature Selection), OR (odds ratio), and CHI2 (Chi-square). These experiments were implemented utilizing four different benchmark datasets in [21]. Based on MICC (Mutually Informed Correlation Coefficient) combining two popular statistical dependence measures namely MI (Mutual Information) and PCC (Pearson Correlation Coefficient), a score-based filter FS approach was presented in [22]. It is known that the performance of generating a framework for machine learning or deep learning is decided using a number of parameters, such as the object’s feature definitions, factors chosen during NN model training evaluation. The stages during image preprocessing for detected or recognized objects are very important. First of all, FS plays a critical role in NN training model optimization.

In the neural-like object detection and classification operations, FS and ranking play a very important role. The potential features of each object can obtain different scores through the FS process. The “N” features can then obtain the best scores. It is completed by calculating the frequency scores of the features in the training set, and then the FS functions and ranking are obtained for the positive and negative samples, respectively. The function contains many factors that must be monitored, detected and classified, some of which turn into other features that may be useless or useful. By removing useless features, the calculation time is reduced to obtain higher performance and further improved accuracy.

In fact, in the traditional FS method, feature extraction is deployed before feeding a learning engine to classify different activities. This is the same as extracting the CSI concept (channel state information) used in wireless communication channels. Based on the previous viewpoint, different activities can be distinguished by analyzing the signal patterns, particularly the dynamics of fine-grained CSI. Hence, the authors in [23] explored wideband Wi-Fi channel information and seamlessly hopped among adjacent channels to create an extended channel. This investigation uses advanced deep learning toward more accurate and robust activity recognition. Additionally, in [24] multimodal medical image fusion, feature-level fusion is accomplished by extracting various presented features to evaluate and implement different pairs of medical image modalities. The results demonstrate that the proposed method improves the quality of the final fused image in terms of the MI (mutual information) and CC parameters. Another study presented in [25] enhanced the visibility of the IR (infrared) night vision images through an efficient histogram processing method that includes histogram equalization and matching. However, it is worth noting that the FS technique from “information” is pretty vital for studying machine learning recognition activities.

## 3. The CC Assignment Scheme

In fact, to effectively measure the degree of divergence between many qualitative random variables when dealing with these random variables, information theory is the best solution. On the other hand, the so-called information theory can provide solutions to the problem of dissimilarity between information uncertainties. In the application of large amounts of data, information theory has theoretical conditions sufficient to support finding the degree of dependence between random variables. Thus, the proposed CC assignment mechanism is derived in this section using the information theory steps.

### 3.1. CC Assignment Algorithm Establishment

As mentioned previously, information theory is the best solution to measure the degree of dependence between random variables. Specifically, the most common user uses the CC (correlation coefficient) between variables to determine the degree of dependence. In other words, the CC is a statistical calculation method for evaluating the similarity between arbitrary random variables. The dependence can determine the strength of the two-way linear connection between two actual random variables approximately. It is known that the correlation amongst the extracted fused and original images is able to be estimated as [26].

(1) $$CC_{n}=\frac{\sum_{u}\sum_{v}\left(X_{i}-\hat{X}\right)\left(Y_{i}-\hat{Y}\right)}{\left\{\left[\sum_{u}\sum_{v}{\left(X_{i}-\hat{X}\right)}^{2}\right]\left[\sum_{u}\sum_{v}{\left(Y_{i}-\hat{Y}\right)}^{2}\right]\right\}}$$

where u and v represent corresponding to the length and width of the original fruit image, while $\hat{X}$ and $\hat{Y}$ are the estimated average values of the $X_{i}$ and $Y_{i}$, respectively.

**Figure 1:**
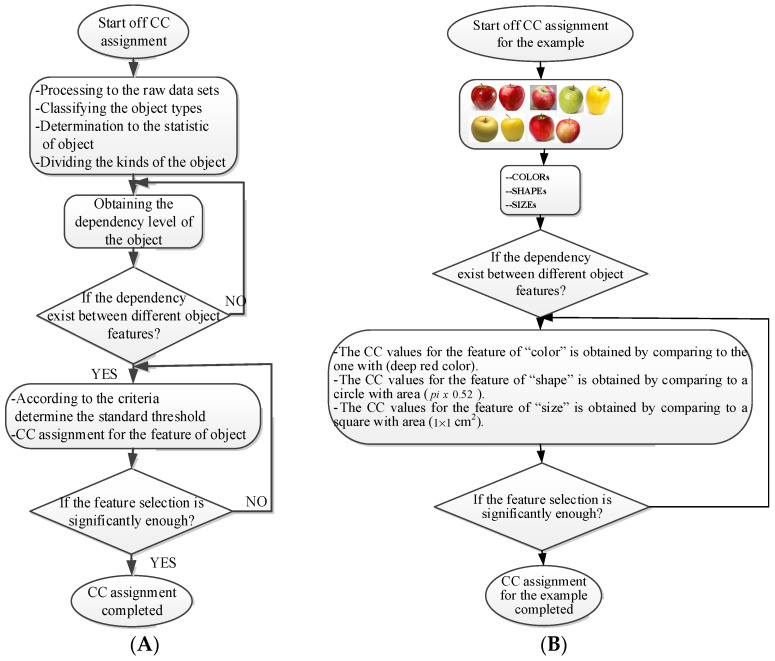
The algorithms for the proposed CC assignment scheme. (**A**) Flow chart of CC assignment; (**B**) Detail flow chart of CC assignment.

Assume that there are n types for one of the classified objects (for examples, fruits, animals, and electronic components, and so on), then $\rho_{n}$ represented as the correlation between *n* types; that is, there are *N* independent random variables, $\rho_{n},\;\;n=1,\;2,\;3\ldots ,N$, with equally CDF (cumulative distribution function) CDF(ρ), and pdf (probability density function) pdf(ρ). Arrange the *n* random variables from small to large into $\rho_{\left(1\right)}\leq \rho_{\left(2\right)}\leq \cdots \leq \rho_{\left(n\right)}$, where $\rho_{\left(r\right)}$,  r=1, 2, 3,…,n is the *r-*th ordered variate. Hereafter, the probability of an event happens between a range, referred as $\rho <\rho_{\left(r\right)}\leq \left(\rho +\delta \rho \right)$, where δ denotes a much little value, and then which can be given as [7]

(2) $$\begin{array}{ll} pdf & \left(\rho <\rho_{\left(r\right)}\leq \rho +\delta \rho \right)\\ & \;=\frac{n!}{\left(r-1\right)!\left(n-r\right)!}{\left[CDF\left(\rho \right)\right]}^{r-1}{\left[1-CDF\left(\rho +\delta \rho \right)\right]}^{n-r}\\ & \;\;\;\;\times \left[CDF\left(x+\delta x\right)-CDF\left(x\right)\right]+O\left({\left(\delta x\right)}^{2}\right) \end{array}$$

where $O\left({\left(\delta \rho \right)}^{2}\right)$ indicts as the probability of a random variable, $\rho_{i}$, located at the duration of (ρ, ρ+δρ]. By dividing the δρ to both sides of an equal symbol and let the δρ approach zero. By substituting r=n into Equation (2) the pdf of the maximum ordered variate for $\rho_{\left(n\right)}$ can be determined as

(3) $$\begin{array}{ll} pdf_{n} & \left(\rho \right)=\frac{n!}{\left(n-1\right)!\left(n-n\right)!}{\left[CDF\left(\rho \right)\right]}^{n-1}\cdot {\left[1-CDF\left(\rho \right)\right]}^{n-n}\cdot pdf\left(\rho \right)\\ & =n{\left[CDF\left(\rho \right)\right]}^{n-1}\cdot pdf\left(\rho \right) \end{array}$$

where (n−n) still kept on the last equation. Accordingly, the random variables $\rho_{n},\;n=1,\;2,\;3\ldots ,N$ discussed in previous equations referred as the CC which is the most popular metrics applied to the literature of PCC (Pearson correlation coefficient) [24]. Certainly, the jpdf (joint pdf) of two or much larger number of ordered random can be extended by following up the same procedures done previously. Therefore, the jpdf corresponding to *k* ordered observations (random variables), $x_{1}\leq x_{2}\leq \cdots \leq x_{k}$, $X_{\left(n_{1}\right)},X_{\left(n_{2}\right)},\ldots ,X_{\left(n_{k}\right)}$, $\left(1\leq n_{1}<n_{2}<\cdots <n_{k}\leq n;1\leq k\leq n\right)$ can be calculated as

(4) $$\begin{array}{ll} f_{n_{1},n_{2},\ldots ,n_{k}} & \left(x_{1},x_{2},\ldots ,x_{k}\right)\\ & =\frac{n!}{\left(n_{1}-1\right)!\left(n_{2}-n_{1}-1\right)!\cdots \left(n-n_{k}\right)!}{\left[F\left(x_{1}\right)\right]}^{n_{1}-1}f\left(x_{1}\right)\\ & \;\;\;\;\times {\left[F\left(x_{2}\right)-F\left(x_{1}\right)\right]}^{n_{2}-n_{1}-1}f\left(x_{2}\right)\cdots {\left[1-F\left(x_{k}\right)\right]}^{n-n_{k}}f\left(x_{k}\right)\\ & =n!\left[\prod_{j=1}^{k}f\left(x_{j}\right)\right]\prod_{j=0}^{k}\left\{\frac{{\left[F\left(x_{j+1}\right)-F\left(x_{j}\right)\right]}^{n_{j+1}-n_{j}-1}}{\left(n_{j+1}-n_{j}-1\right)!}\right\} \end{array}$$

where each of the employed symbols are defined completely. Hence, just as the result of the joint object features statistical characteristics, a certain standard threshold must be set in advance for all different characteristics. The designation of the CC proposed in the current article is based on the degree of difference between the object characteristics (selective features) and the standard threshold. Therefore, a diversified FS is produced in the special processing stage, and the probability of datasets occurring with high-dimensional inappropriateness is reduced. Moreover, the algorithm for completion the CC assignment is according to the critical benchmark involves the flows shown in Figure 1A. In the algorithm, the CC assignment mainly determines SC to classify the object types, such as the “color”, “shape”, and “size” features of an APPLE, as arranged in this study [27]. An example is shown in Figure 1B used to deeply explain the CC assignment procedures. Hereafter, there are nine different types of apples provided to the algorithm for the CC assignment work. The dependency judgement to be completed follows up the three features occurring in all of the provided apples’ photos.

### 3.2. Feature Extraction Algorithm and CC Assignment Scheme

**Figure 2:**
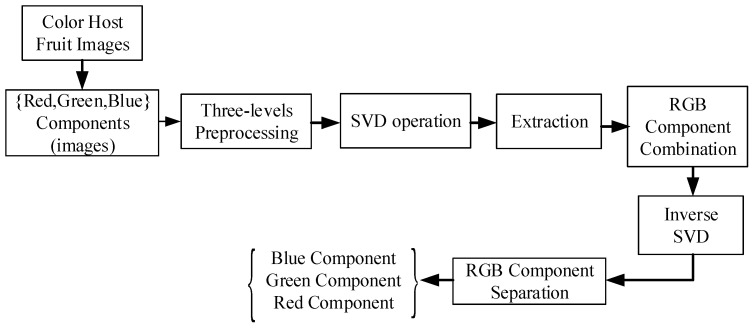
Flowchart of the extraction process of the SVD algorithm.

Strictly speaking, feature extraction is the most crucial stage before the aforementioned activity for completing the CC assignment scheme. According to the authors’ knowledge, it is known that the proposed schematic of the CC assignment is a brand new analysis. The feature extraction process steps from the host color fruit image are shown in Figure 2, and it can be summarized as follows:

1. The colored fruit images are separated into three RGB components (Red, Green, and Blue).
2. Three levels of the SVD (singular value decomposition) are repeatedly separated for each of the RGB color fruit image components.
3. Three CC levels are applied to the resulting sub-bands to obtain their respective correlated coefficient matrices.
4. The SVD is performed on the approximation and all detailed components of the third level coefficients of each colored fruit image RGB component.
5. RGB component matrices of the colored fruit image are computed.
6. After the three extracted fused colored fruit images, an encrypted and fused fruit image is obtained, repeatedly.
7. Correlation amongst the extracted fused and original images is estimated.
8. Correlation amongst the extracted three gray-scale fruits and original images is estimated. The high values mean the presence of the fruit with good quality.

In fact, the feature extraction algorithm is proposed in the current article, where the technique is mainly dependent on the features of SVD. The main aim of this proposed technique is to achieve reliable robustness for the color images. Accordingly, the SVD is a numerical technique which decomposes the input data into expected submatrices [10]. Basically, a diagram matrix whose diagonal elements have singular values will be obtained after decomposition of the input image. Assume that A is the input signal, U and V denote the left and right singular vector matrixes, respectively, and these singular values are corresponding the energy of the signal. The S will be presented as the diagram matrix which is determined as

(5) $$A=USV^{T}$$

where ${\left(\cdot \right)}^{T}$ is the matrix transformation. Based on the SVD technique and the steps shown in Figure 2, the dark or light of color can be distinguished as seen in the descriptions next.

**Figure 3:**
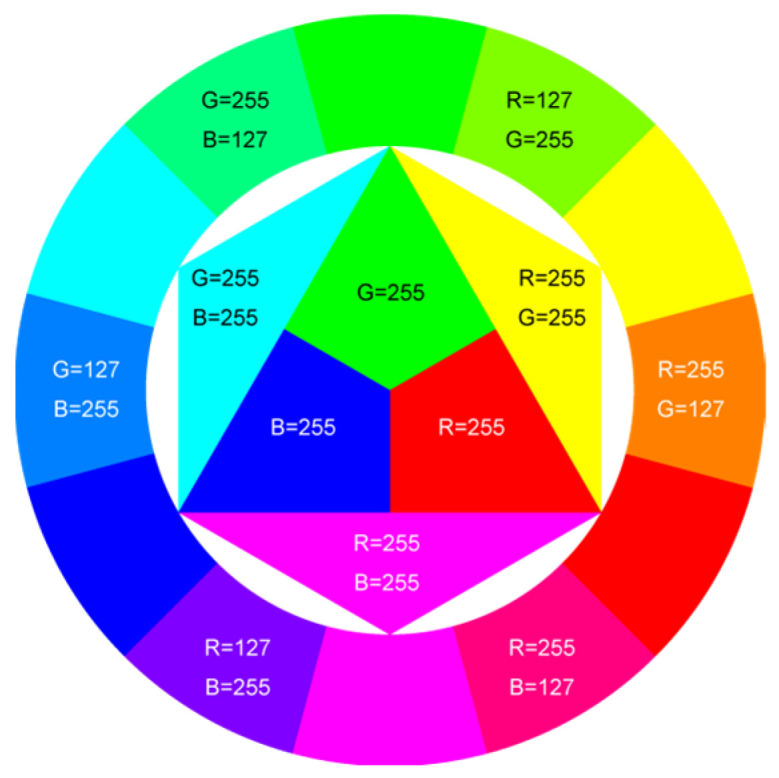
The color value map of different colors.

In Table 1, different types of apples are used to show examples of obtaining the CC values. The nine types of apples include “Apple red 1”, “Apple red 2”, “Apple pink lady”, “Apple granny smith”, “Apple golden 3”, “Apple golden 2”, “Apple golden 1”, “Apple crimson snow”, and “Apple Braeburn” and their attached photos. Among them, the article uses three different selected features, “color”, “shape”, and “size”, to show the distinct CC values of different apple types. The apple FS for these three is only used as an example. Of course, other features of different identification objects are also able to be added during the procedure of feature extraction. Therefore, it is critical that points out how to specify the CC values of these three features; that is, a reasonable mechanism must be established. For example, the assessment with different apple colors: first of all, this example uses the dark or light of a color which is as shown in Figure 3. On the other hand, the color value mapping to the color demonstrated in Figure 2 is the widely used method.

Hereafter, according to the equation shown in Equation (1), an example uses the weighting formula of RGB with three original colors to specify the CC value for the FS. That is, the proposed assignment scheme to obtain the CC value can be sub calculated as

(6) $${\mathrm{CC}}_{v}=\rho =\left[\left(\alpha \cdot R+\beta \cdot G+\gamma \cdot B\right)/k\right]{+b}_{i}$$

where *k* is the summation of original colors, that is, k=R+B+G, α, β, and γ are weighting values for R, G, and B, respectively, and the $b_{i}$ denotes a bias error value caused by the human’s sight. The bias error is assigned as 0.05 for the reason of simplicity. Table 1 shows the corresponding values obtained by Equation (6) mapped to the CC values.

First, the “middle value” is used as the standard threshold value for “red” color, and as the basis for the assigned CC value under restricted environment of FS. As far as red is concerned, there will be many color shade differences; thus, the CC for the color features is specified according to the degree of dissimilarity between the red color differences. For example, the CC designated number of “Apple pink lady” is determined as ${\mathrm{CC}}_{v}=\left[\left(0.9\times 255+0.7\times 255+0\right)/(255+255+0)\right]\pm 0.05$; thus, the CC value ${\mathrm{CC}}_{v}=0.8\;\pm \;0.05$ is assigned to it. The next two features consider the “shape” and “size” features of an apple. The CC values for the “shape” and “size” features are decided by comparing to a circle with a unit area ($\pi \times 0.5^{2}$) that has the (1 cm) radius and the area of a square that has the (1 cm) side length, respectively. Thus, the CC values shown in Table 2 are easily filled. Onwards, let us give one more illustration for the CC assignment operation, e.g., the CC size value of 0.7±0.05 for “Apple golden 2” is calculated as follows, ${\mathrm{CC}}_{v}=\left[\left(0\times 255+0.9\times 255+0.5\times 255\right)/1\right]\pm 0.05$.

## 4. Results and Discussions

After completing the theoretical deduction for the CC assignment mechanism, three models are developed based on CNN-type NNs, including AlexNet, GoogLeNet, and ResNet101. These models will be adopted to perform actual calculation tests and analysis. The results are discussed and analyzed in this section. Moreover, this paper evaluated the proposed CC assignment scheme using the standard datasets with 120 different kinds with a total of 20,860 fruit image datasets collected from AlexNet, GooLeNet, and ResNet101. The results are employed to verify that the accuracy rate can be improved by reducing strict FS. The preprocessing of the gathered photos is also going to be discussed briefly in the following subsection.

### 4.1. The Deployment of Experiments

The exploration to collect the pictures of apple images is firstly presented as mentioned previously. In fact, the images were gathered by picturing the apples while they were circled by a rotating motor. Fruits were scaled to fit a 100 × 100 pixel image. Other datasets (such as MNIST) use 28 × 28 images. First, the experiment will discard the photos that are not likely to help our analysis. For example, the training work makes the assumption that the images with “broken fruit” will not be very useful in our task, because we need a “complete fruit” as shown in the photos which will benefit calculating the CC value as discussed in Section 3. With such thinking, we discard the fruit images with the features of “broken”, “rough”, and “too small (big)”. The conditions described above are likely constraints on the application of the proposed CC assignment scheme; however, the presented model struggles with diversity of different fruits even other objects.

**Figure 4:**
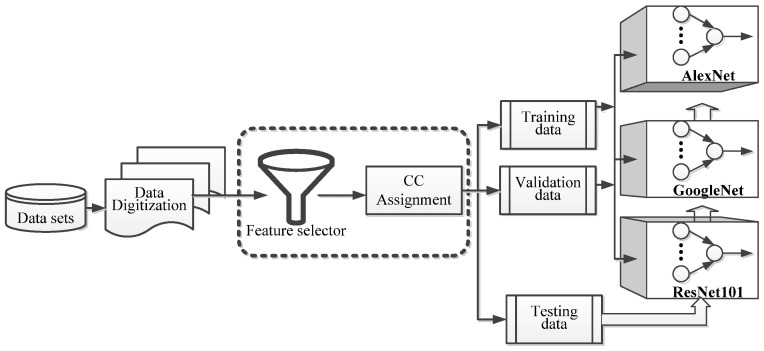
The block diagram of experiments.

Figure 4 shows the block diagram of experiments deployed in this subsection. The photo data was digitized after they were collected. There is a feature selector designed to extract the features that accompany the recognized image. The CC assignment scheme is applied to identify the CC values for these features. The model for NN with the data after being assigned CC values and follow up training and validation activities is completed.

For simulation purposes, the validity of the proposed hybrid features for fruit images framework using CC assignment techniques is evaluated and confirmed. Several different colored apple images were used in our evaluation tests. The simulations were carried out using MATLAB 2020a on a Windows 10, 64-bit operating system with an AMI**^®^** Core™ i7-7700HQ CPU @2.80 GHz with 16 GB RAM. In all experiments, the quality of the extracted different kinds of apple images were assessed through the CC assignment.

The model is certified using testing data with the accuracy rate calculated using the three NNs separately. Particularly, the square area with a dotted line significantly expresses the most critical contributions of the proposed scheme. The issues mentioned previously include the feature selector design and the CC assignment algorithm discussed in Section 3.

### 4.2. Introduction to the Adopted NN Models

**Figure 5:**
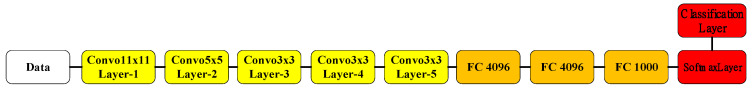
The block diagram of AlexNet model.

**Figure 6:**
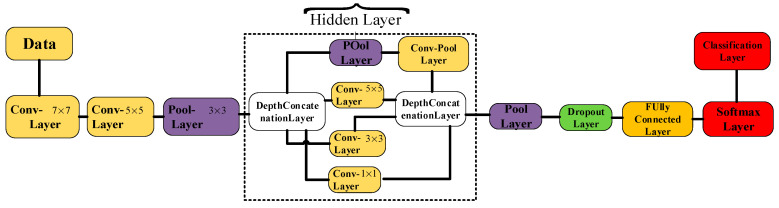
Block diagram of GoogLeNet model.

**Figure 7:**
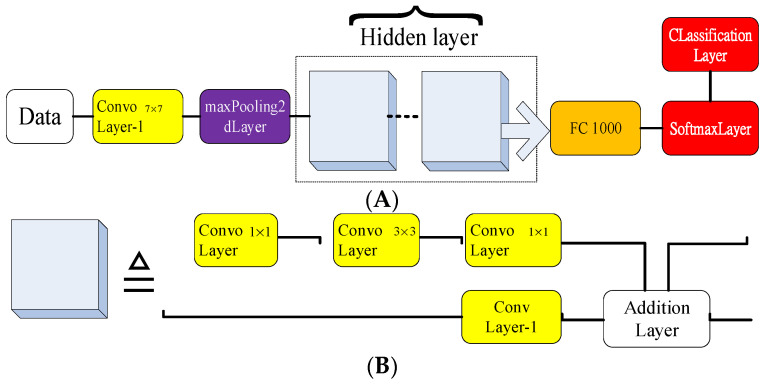
(**A**) Block diagram of ResNet101 model; (**B**) Block diagram of the hidden layer.

Basically, the three most popular NNs, i.e., AlexNet, GoogLeNet, and ResNet101, developed based on the CNN algorithms, were applied when verifying the proposed schemes. Accordingly, the three adopted NN models are shown in Figure 5, Figure 6 and Figure 7, respectively. The AlexNet shown in Figure 5 has five convolutional layers constructed with different numbers of neurons. The fully forward propagation layers are deployed before the Softmax layer, which plays the role of activation function. The final classification results are decided next.

The GoogLeNet model with the hidden layer is constructed using two deep concatenation layers after the input layer. The output classification layer follows the fully connected and softmax layer. A full block diagram of the GoogLeNet model is shown in Figure 6.

The block diagram of the ResNet101 model is shown in Figure 7A,B. A variate number is assigned to the hidden layer to adjust the accuracy rate for the training stage [25]. The CNN model deployed content belongs to the hidden layer as shown in Figure 7B.

### 4.3. The Results and Discussion

In fact, the performance metrics should be decided before discussing the outcomes from the experiments. In this investigation, the training accuracy, precision, and training loss are used to evaluate the proposed methods. When real and assigned class labels are compared, it results in *TP* (true positive), *TN* (true negative), *FP* (false positive), and *FN* (false negative). The performance metrics evaluation is formulated as follows [28,29,30]:

(7) % of Accuracy=[(TP+TN)/(TP+TN+FP)]×100%

(8) % of Precision=[(TP)/(TP+FP)]×100%

(9) % of Loss=1-(% of Accuracy)

The FS assessment is definitely one way to solve the burden of an overfitting, high volume of dimensional data during the computational algorithm. Therefore, it is worth discussing how the FS method is employed to overcome the performance degradation caused by those interfering factors. The proposed algorithm in the current article is a feasible way which has been verified by the results discussed in this subsection.

There are three popular NN frameworks, AlexNet, GoogLeNet, and ResNet101, deployed to the proposed scheme to approve the outcomes from the feature computing. In addition, the three different solvers include “Sgdm (Stochastic Gradient Descent)”, “Rmspron (Root Mean Square Prop)”, and “Adam (Adaptive Moment Estimation)”, separately applied to the NN models. The toolbox provides the necessary details to develop the NN model training schemes coming from the work of [18]. For example, the activation function, the pooling layer, fully Connected Layer, Normalization Layer, and so on. In Algorithm 1, a brief pseudo code manuscript is illustrated for the NN training process for the photos. The program implementation is shown in Algorithm 1. In fact, many basic functions were utilized to complete the training activity. The packet library for training the datasets deployed in the experiment is adopted as the “trainingOptions(.)” function. The “options” depend on which NN framework is used currently. On the other hand, the dedicated three NN frameworks are assessed to play the roles of experiment, and three optimizers are also listed, Sgdm, Rmspron, and Adam, independently. Furthermore, the utilized super parameters are assumed as MiniBatchSize = 2280, MaxEpochs = 50, InitialLearnRate = 1 × 10^−4^, and many other different items are following.
**Algorithm 1** A Brief Pseudo Code Function for the NN Training1:*////Output*2:**function** [imds,layers,options] = Experiment1_setup1(params)3:*/////Load Image Data*4:**dataFolder**5:     fullfile(‘C:\Users\H738\Desktop\XXXS\Experiment1\CH7_1\TEST’);6:     imds = imageDatastore(dataFolder, …7:        ***if*** ‘IncludeSubfolders’,true, …8:             ‘LabelSource’,‘foldernames’);9:             numTrainingFiles = 0.1;10:             [imds,imdsValidation] = splitEachLabel(imds,numTrainingFiles);11:        ***endif***
12:*////Define Network Architecture*13:        ***switch*** params.Network14:            ***case*** “alexnet”15:                 load(‘AlexNet’,’layers_1’)16:///parameters announce 18:                 (inputSize; imds; imdsValidation;imdsValidation);19:                 layers = layers_1;20:            ***case***“googlenet”21:                 load(‘GoogLeNet’,’lgraph_1’)22:///parameters announce23:                 (inputSize; imds; imdsValidation;imdsValidation);;24:                 layers = lgraph_1;25:            ***case***“resnet101”26:                 load(‘Resnet101’,’lgraph_2’)27:///parameters announce 28:                 (inputSize; imds; imdsValidation;imdsValidation);;29:                 layers = lgraph_2;30:            ***otherwise***
31:                 msg = [‘Undefined network selection.’ …32:                                 ‘Options are “default” and “googlenet” and “resnet101”.’];33:                 error(msg);34:***end***35:*///Specify Training Options*36:         options = trainingOptions(params.Solver, …37:         ‘MiniBatchSize’,2280, …38:         ‘MaxEpochs’,50, …39:         ‘InitialLearnRate’,1 × 10^4^, …40:         ‘Shuffle’,’every-epoch’, …41:         ‘ValidationData’,imdsValidation, …42:         ‘ValidationFrequency’,5, …43:         ‘ExecutionEnvironment’,“cpu”,…
         ‘Verbose’,true, …
                 ‘plots’,‘training-progress’);

The evaluation quality metrics are presented in Equations (7)–(9). The outcomes obtained from the code implemented without FS carry out the three NNs as demonstrated in Table 3. That is, the feature selector is ignored in the data training and testing scenario. Photos for different kinds of apple are used to obtain the implementation from the database in [28], in which the fruits are currently referred to as NFS (non-function selection) categories. This means that the images first processed do not have FS activity. The test-to-training data ratio number is seven to three (7:3).

It is valuable to observe that the lowest accuracy rate occurs at the NN of AlexNet. Sgdm, Rmspron, and Adam solver produced accuracy rates of 30.75%, 40.0%, and 45.97%, respectively. Even the highest training loss rate emerged in such cases; that is, they correspond with a 1.94, 1.64, and 1.75 loss rate. The phenomena could be explained from the viewpoint of the “Elapsed Time” which always takes the least execution time. The NN of the AlexNet model was constructed with a concise structure, for example, a less fully connected layer. Alternatively, GoogLeNet was established with 22 NN layers. In contrast, the AlexNet contains a total of eight layers, i.e., the convolution layer is assigned to five layers, two for the maximum pooling layer, and one fully connected layer [31].

Moreover, it is worth noting that the ratio of test-to-training affects the percentage of accuracy rate and training loss. The results from Table 3 show that the test-to-training ratio is demonstrated for AlexNet NN only, because the limitation of editorial size is considered. It is seen that the larger the training data number, the better the performance accuracy rate. That is, the accuracy rate is equal to 40.87% and 30.75% when the ratio of test-to-training is 3:7 and 7:3 with Sgdm solver, respectively. Similarly, for the case illustrated with Rmspron, the accuracy rate is equal to 80.70% and 40.00% for the ratio of test-to-training corresponding to 3:7 and 7:3.

Furthermore, the factor of elapsed time is also shown in Table 3. It is reasonable that the elapsed time is always incremental, while the executing case has a larger number of training data, that is, the time wasted is usually much longer when there is a huge number of training data size. For instance, the elapsed time is 34 min for 3:7 case of Alex NN with Sgdm Solver, and the elapsed time is shorter than that which executes the situation with a test-to-training ratio of 7:3

Table 4 shows a crucial point in the comparison with other work [32], which gets a classification accuracy of 98.88% when applying the proposed Pure CNN (PCNN) with texture features. It is easily observed that the current proposed CC assignment outperforms with 99% classification accuracy.

**Figure 8:**
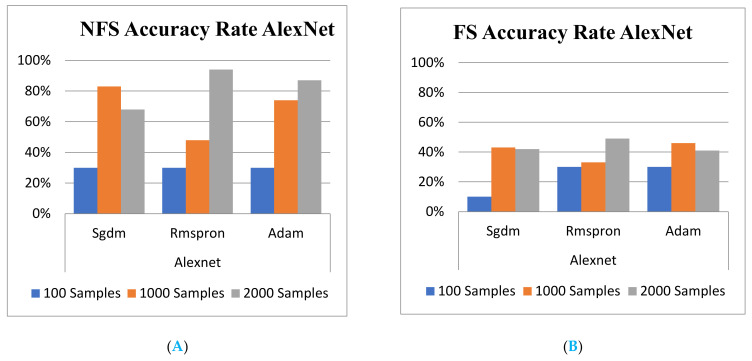
Training Accuracy rate for GoogLeNet. (**A**) with FS; (**B**) without FS.

Table 5 and Table 6 show the total training and validation accuracy rate outcomes from the three NN learning models with and without NFS, respectively. Accordingly, the training accuracy rate outcomes shown in Table 5 and Figure 8A,B dictated that a significant difference occurs in the ResNet 101.

**Figure 9:**
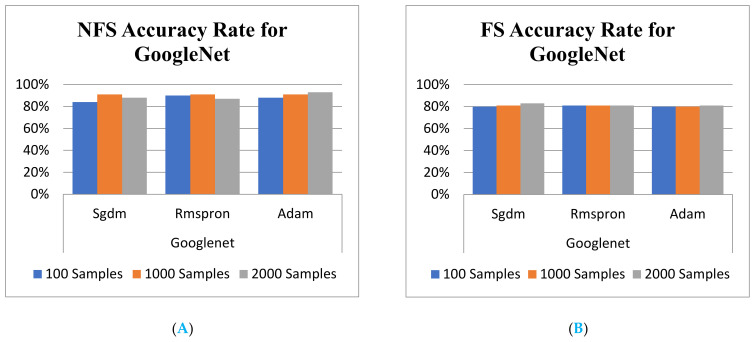
Validation Accuracy rate for GoogLeNet. (**A**) with FS; (**B**) without FS.

It is known that the results from the validation accuracy rate, shown in Table 6 and Figure 9A,B, do not completely outperform those of the NFS after NN training. However, it is definitely dependent on the NN framework and the kinds of solver. In addition, the validation accuracy demonstration rate for one of the NNs, GoogLeNet training model, with and without NFS are shown in Figure 9A,B, respectively. For grasping the significant promotion has breakthrough caused by the CC assignment propose in this article. There has a brief comparison between the results from the scheme presented in Figure 9 with that is shown in Table 1 and Table 2 of [32]. Accordingly, it is easy to see that the classification accuracy is always less than 92% under any case of # of feature (20 to 240). However, the outcome of accuracy rate shown in Table 4 can arrive at 99% with a 100 sample size for GoogLeNet and ResNet101, which is enough to recognize that the performance of the proposed scheme has significantly outperformed the traditional methods.

Finally, the trust level for the proposed schemes is worth demonstrating in order to prove the trustable validation. Hence, the testing activities are carried out by feeding different feature images all randomly specified with FS and without FS into the trained models for recognition. The comparison of training and testing accuracies of various classification learner models depicts shown in Figure 8 and Figure 9 with histogram, respectively. Comparison comprises of Euclidean distance and mutual information as well as different model accuracies using NFS and FS data obtained using the proposed method. The highest testing accuracies are found in GoogLeNet with Rmspron solver, while the lowest testing accuracies are found in AlexNet learner.

By using the same procedure for validation, the three NN trained models were employed to assess the testing activity performance. The sequential results from the testing activity are demonstrated in Table 7 where the feed image and possibility outcome are shown on the left and right-hand sides, respectively. The rectangular block located at the right-hand side has a bar that shows the likely fruit name located at the left-hand side. The X-Axis and the Y-Axis in the bar chart represent the likely probability and characteristics (fruit name, color, size, or smooth) of the outcome. For example, the fruit photo shown in the first column and first row of Table 7 is labeled with the name “Apple Red 1”, since it has the longest bar length after the recognition is completed by AlexNet NN. Even the photos of rotten apples shown in the “Sgdm” case, the recognition is still able to be certainly discriminated. In summary, there are many easily noted outcomes explained and listed below for editorial size reasons.

A. The characteristics of each tested fruit can be accurately recognized if the feature is determined with FS.
B. It is known that the results from the testing activity have the higher probability occurred both at the GoogLeNet and ResNet101 frameworks. This especially happens in GoogLenet. This phenomenon copes with that previously discussed in the validation procedure. That is, let us recall the validation activity mentioned previously with a significant accuracy rate when the Google Net model is applied.
C. The CC exists between the selective features and is certainly able to play as a critical parameter that affects the recognition accuracy rate with NN computing.

## 5. Conclusions

In order to overcome the NN limitation of data overfitting, high-dimensional data, and feature selectivity, this paper proposed a CC assignment mechanism for the distribution of different kinds of objects. On the other hand, in order to pursue the solution utilized in solving the restrictions of NN computing and adaptively facilitate the traditional FS categories, the CC (correlation coefficient) assignment scheme is developed in the current article. The CC value is assigned to different features between categories depending on their characteristics. The identification of environmental conditions was expanded. In other words, this research adopted the specifying CC assignment method to classify different object categories to obtain higher feature dependence (FS), especially after the adaptive CC assignment is applied. Feature selectivity can amplify the degree of difference between object features. Finally, through jpdf derivation of the correlation elements, the theoretical basis for the CC configuration was obtained. In the mechanism, CNN-based AlexNet, GoogLeNet, and ResNet101 were used to perform the training, testing, and actual validation activities. The results from the evaluated performance of these models were compared with the existing traditional method results without CC designation. It was found that the training framework using the proposed adaptive CC assignment in this article performs better than the outcomes from the traditional methods. In future works, much more different NN frameworks are going to be explored and compared with each other by using the CC assignment scheme, with the aim of simplifying and facilitating the autonomous driving.

## Figures and Tables

**Table 1:** The mapping table for original CC to the final CC values.

$0<{\mathrm{CC}}_{v}<88$	$89<{\mathrm{CC}}_{v}<176$	$177<{\mathrm{CC}}_{v}<255$
$0<{\mathrm{CC}}_{v}<0.2$	$0.3<{\mathrm{CC}}_{v}<0.6$	$0.7<{\mathrm{CC}}_{v}<0.9$

**Table 2:** Example of CC values for different kinds of apples.

Name of Apples		Apple Red 1	Apple Red 2	Apple Pink Lady	Apple Granny Smith	Apple Golden 3	Apple Golden 2	Apple Golden 1	Apple Crimson Snow	Apple Braeburn
Photos		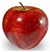	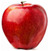	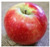	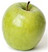	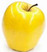	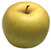	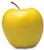	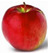	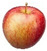
**CC** **VALUEs** **(*ρ*)**	C O L O R s	The CC values for the feature of “color” is obtained by comparing to the one with (deep red color).								
		0.9 ± 0.05	0.9 ± 0.05	0.8 ± 0.05	0.1 ± 0.05	0.1 ± 0.05	0.1 ± 0.05	0.1 ± 0.05	0.8 ± 0.05	0.6 ± 0.05
	**S** **H** **A** **P** **E** **s**	The CC values for the feature of “shape” is obtained by comparing to a circle with area (π × 0.5^2^).								
		0.7 ± 0.05	0.9 ± 0.05	0.8 ± 0.05	0.7 ± 0.05	0.4 ± 0.05	0.8 ± 0.05	0.5 ± 0.05	0.8 ± 0.05	0.7 ± 0.05
	**S** **I** **Z** **E**	The CC values for the feature of “size” is obtained by comparing to a square with area (1 × 1 cm^2^).								
		0.7 ± 0.05	0.9 ± 0.05	0.7 ± 0.05	0.8 ± 0.05	0.8 ± 0.05	0.7 ± 0.05	0.9 ± 0.05	0.9 ± 0.05	0.9 ± 0.05

**Table 3:** Accuracy/validation rate results without FS to carry out the three NNs.

Network	Elapsed Time	Solver	Accuracy Rate %	Training Loss	Validation Accuracy%	Validation Loss	Ratio of Test-to-Training Data
AlexNet	34 min	Sgdm	40.87	1.63	53.59	1.99	3:7
	56 min		30.75	1.94	50.59	1.92	7:3
GoogLeNet	4 h 25 min	Sgdm	88.20	0.37	68.25	0.87	7:3
ResNet101	3 h 50 min	Sgdm	98.40	0.11	61.70	1.21	7:3
AlexNet	35 min	Rmspron	80.70	0.58	70.15	0.99	3:7
	49 min		40.00	1.64	59.49	1.65	7:3
GoogLeNet	3 h 51 min	Rmspron	99.00	0.00	84.82	0.46	7:3
ResNet101	23 h 12 min	Rmspron	98.20	1.37	87.44	0.35	7:3
AlexNet	32 min	Adam	69.92	0.87	53.94	1.67	3:7
	1 h 7 min		45.97	1.75	65.91	1.42	7:3
GoogLeNet	5 h 40 min	Adam	99.80	0.01	92.34	0.22	7:3
ResNet101	23 h 51 min	Adam	97.60	2.15	90.13	0.32	7:3

**Table 4:** A results comparison between the current proposed and othre work.

	Dataset	NN Framework	Methods	Conditions	Classification Accuracy
[32]	Fruits-360 (Apple)	Pure-CNN	Non FS	7 convolutional layers	98.88%
Proposed	Fruits-360 (Apple)	ResNet101 (CNN-based)	With CC assignment	CC-Assignment	May over 99%

**Table 5:** Total accuracy rate outcomes for training from the three NN frameworks.

Neural Network	Solver	Color (Red)			Appearance			Size			With/Without Feature Selection (FS/NFS)					
		Dark	Med.	Light	Smooth	Little	Rough	Large	Med.	Small	NFS Accuracy Rate %			FS Accuracy Rate %		
		Accuracy Rate %									Sample Size					
											100	1000	2000	100	1000	2000
Alexnet	Sgdm	71.0	84.0	710.	41.0	70.0	40.0	54.0	55.0	40.0	30	83	68	10	43	42
	Rmspron	88.0	70.0	88.0	40.0	55.0	33.0	33.0	54.0	40.0	30	48	94	30	33	49
	Adam	85.0	88.0	95.0	51.0	43.5	96.0	94.0	52.0	58.0	30	74	87	30	46	41
GoogLenet	Sgdm	88.0	85.0	88.0	88.0	33.0	33.0	33.0	33.0	33.0	99	99	99	91	90	91
	Rmspron	96.0	99.0	98.0	94.6	66.0	45.0	90.0	44.7	97.0	99	99	99	92	90	92
	Adam	65.0	99.0	97.0	95.0	64.0	66.0	97.0	85.0	55.0	99	99	99	87	90	87
Resnet101	Sgdm	99.0	99.0	99.0	99.0	99.0	37.0	41.0	33.0	33.0	99	99	99	90	99	99
	Rmspron	99.0	98.0	98.0	99.0	99.0	41.0	46.0	57.0	47.0	99	99	99	90	99	99
	Adam	99.0	99.0	99.0	99.0	99.0	33.0	33.0	34.0	51.0	99	98	98	89	88	80

**Table 6:** Total accuracy rate outcomes for validation from the three NN frameworks.

Neural Network	Solver	Color (Red)			Shape			Size			With/Without Feature Selection (FS/NFS)					
		Dark	Med.	Light	Smooth	Little	Rough	Large	Med.	Small	NFS Accuracy Rate %			FS Accuracy Rate %		
		Accuracy Rate %									Sample Size					
											100	1000	2000	100	1000	2000
Alexnet	Sgdm	33.0	33.0	33.0	33.0	33.0	33.0	33.0	33.0	33.0	13	83	83	49	72	80
	Rmspron	33.0	42.0	33.0	33.0	45.0	33.0	33.0	54.0	40.0	52	48	48	60	75	61
	Adam	50.0	42.5	95.0	58.0	43.5	96.0	94.0	52.0	58.0	43	74	74	79	75	76
GoogLeNet	Sgdm	34.0	33.0	33.0	34.0	33.0	33.0	33.0	33.0	33.0	84	91	88	80	81	83
	Rmspron	96.0	37.0	59.0	94.6	66.0	45.0	90.0	44.7	97.0	90	91	87	81	81	81
	Adam	65.0	55.0	58.0	65.0	64.0	66.0	97.0	85.0	55.0	88	91	93	80	80	81
Resnet101	Sgdm	58.0	33.0	33.0	45.0	36.0	37.0	41.0	33.0	33.0	47	50	50	67	66	67
	Rmspron	37.0	57.0	45.0	37.0	45.0	41.0	46.0	57.0	47.0	93	94	94	89	75	81
	Adam	33.0	33.0	58.0	33.0	33.0	33.0	33.0	34.0	51.0	99	98	98	89	88	80

**Table 7:** The sequential results from the testing activity.

NN	Solver		
	Sgdm	Rmspron	Adam
AlexNet	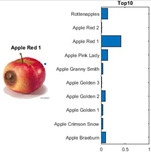	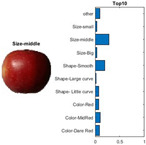	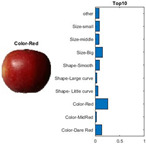
GoogLeNet	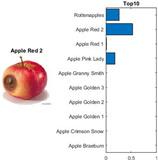	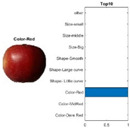	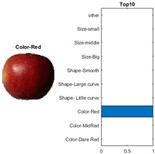
ResNet101	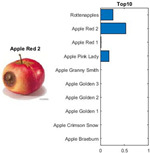	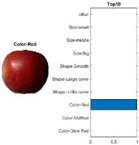	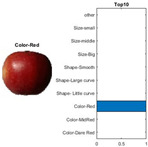

## Data Availability

Not applicable.
